# Thermostable Enzyme Variants in the Lower Mevalonate Pathway Improve Isoprenoid Production by Cell-Free Biocatalysis

**DOI:** 10.1021/acssuschemeng.5c03763

**Published:** 2025-08-06

**Authors:** Sylvia A. Sarnik, Mia R. Martinsen, Tyler P. Korman, Daniel K. Schwartz, Joel L. Kaar, Yannick J. Bomble

**Affiliations:** † Department of Chemical and Biological Engineering, 1877 University of Colorado, Boulder, Colorado 80309, United States; ‡ Biosciences Center, National Renewable Energy Laboratory, Golden, Colorado 80401, United States; § Department of Chemical Engineering, 3557 Colorado School of Mines, Golden, Colorado 80401, United States; ∥ Exozymes, Inc., Monrovia, California 91016, United States

**Keywords:** cell-free biocatalysis, enzyme stability, isoprenoid, mevalonate pathway, thermophilic pathway, limonene

## Abstract

Cell-free biocatalysis is a rapidly evolving field with great potential for sustainably producing valuable chemicals. Some challenges in cell-free biocatalysis include reaction longevity, enzyme stability, and the cost of the biocatalysts. Here, the challenge of enzyme instability was addressed by employing thermophilic enzymes to improve the productivity of the lower mevalonate pathway, using limonene as an example isoprenoid product. The Classical mesophilic mevalonate pathway was compared to a newly assembled set of thermophilic enzymes comprising the Archaea I mevalonate pathway. The thermophilic pathway enzymes were thermostable to at least 60 °C and exhibited a 6× longer operating lifetime at 22 °C. Thus, despite lower initial activity rates at ambient temperature, the thermophilic pathway was longer-lived and resulted in a more productive cell-free reaction overall, achieving 1.7× higher yield of limonene compared to using enzymes from mesophiles. Moreover, the thermostable pathway retained activity for longer with the challenge of solvent exposure, namely, ethanol and isoprenol, which broadens the scope of accessible substrates and/or products in cell-free reactions employing this pathway. Altogether, we showed significant improvement in the stability and productivity of the lower mevalonate pathway, which will enable more efficient cell-free biosynthesis of isoprenoid products. This represents a valuable strategy to increase the robustness of cell-free systems by carefully sourcing biocatalysts from thermophilic organisms, which have proven to be resilient to challenges unique to cell-free reaction systems.

## Graphical abstract

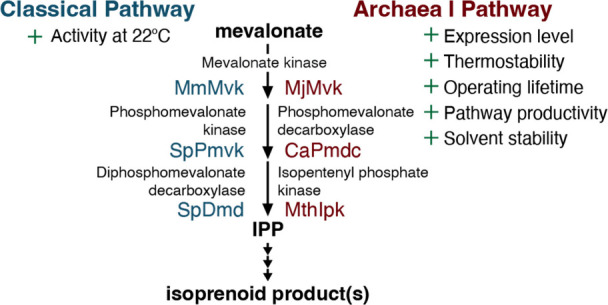

## Introduction

Biocatalysis for the production of chemicals is a rapidly growing field with many diverse applications, ranging from pharmaceuticals to fuels. Enzymes catalyze complex molecular conversions and produce highly specific products, making them an ideal tool for sustainable chemistry, avoiding energy intensive reactions or toxic chemicals often employed in chemical conversions.^1^ Broadly, biocatalysis addresses green chemistry principles of minimizing waste and toxic materials, as well as enhancing energy efficiency, as outlined by Sheldon and Woodley.^2^ Biocatalysis is an attractive approach for producing isoprenoids, including terpene molecules, which are highly valued for their diverse applications, such as anticancer pharmaceuticals and clean-burning, sustainable aviation fuel additives (Figure S1).^3–5^ Currently, isoprenoids are extracted from biomass, which requires energy-intensive processes.^6–9^ Chemical synthesis requires toxic reactants and has proven difficult for these complex molecules.^10–13^ In the last two decades, biosynthesis of these molecules *in vivo* has shown promise. Metabolic engineering has harnessed enzymes to conduct complex chemistries in a sustainable way.^14^ In some cases, fermentations were able to achieve titers over a few g L^–1^.^15–21^ Unfortunately, *in vivo* isoprenoid production is limited by competing metabolic pathways, the complexity of pathway engineering, and the inherent toxicity of isoprenoid precursors—such as isopentenyl pyrophosphate and dimethylallyl pyrophosphate—as well as products like terpene molecules.^22–25^ Cell-free biocatalysis is a promising alternative that addresses these limitations while preserving the environmentally friendly advantages of biocatalysis.^26,27^

In cell-free biocatalysis, enzymes in a pathway are individually produced and then reconstituted in a defined ratio to perform desired reactions *in vitro*. The cell-free approach has demonstrated high productivity in several instances. Even toxic chemicals such as 1,3-butanediol, limonene, and isobutanol can be produced at high titers, achieving 7.7 g L^–1^, 15 g L^–1^, and 275 g L^–1^, respectively.^28–30^ Despite its many advantages, cell-free biocatalysis has its own drawbacks; one major limitation is the operating lifetimes of the enzymes, which are inherently unstable and deactivate over time, especially in the presence of solvents or denaturing products. In contrast to microbial biocatalysis, where metabolism continuously recycles enzymes, in cell-free systems any loss of activity of the initial input enzymes results in decreased productivity of the pathway.^31,32^ Most published cell-free reaction systems operate for less than 24 h,^33,34^ with the longest being 7 days.^29^ Short operating lifetimes increase the economic burden and limit sustainability of cell-free systems by necessitating higher biocatalyst production. An ideal biocatalyst would retain activity for longer to achieve higher product titer in batch-type processes, and even permit recycling for multiple production runs. Alternatively, continuous production systems, such as flow reactors, would be enabled. These improvements, if realized, have the potential to increase productivity while reducing costs, energy use, waste, and the use of toxic compounds.

This study addresses the challenge of enzyme instability by sourcing enzymes from thermophilic organisms, which are generally regarded to be more stable, with the objective of enhancing robustness and productivity of the lower mevalonate pathway toward isoprenoid production. Thermophile-derived enzymes have evolved superior stabilizing characteristics such as dense hydrophobic core packing, increased intermolecular interactions, and disulfide bridges to retain folded structure and catalytic activity at high temperatures.^35,36^ The Ohtake group has pioneered the use of thermophile-derived enzymes for cell-free pathways, with success in numerous applications ranging from n-butanol to Coenzyme A production.^37–39^ Building upon their foundational work, we compared thermophilic and mesophilic cell-free mevalonate pathways to investigate whether enzyme thermostability enhances operational longevity and isoprenoid productivity.

Numerous pathways have evolved for the biosynthesis of isopentenyl pyrophosphate (IPP) and dimethylallyl pyrophosphate (DMAPP), the critical building blocks for isoprenoids. Plants and bacteria have developed the DXP (or MEP) pathway. Eukaryotes and Archaea utilize versions of the mevalonate pathway, the Classical (or canonical) and Alternative (or Archaea) pathways, respectively, for producing isoprenoids.^40–43^ Intriguingly, several branching Archaeal mevalonate pathways have evolved including the Alternative Mevalonate 1 (Archaea I) discovered by Dellas et al. in 2013, the Alternative Mevalonate 2 (Archaea II) discovered in *Thermoplasma acidophilum* by Vinokur et al. in 2014, and the Alternative Mevalonate 3 (Archaea III) pathway identified in Aeropyrum pernix by Hayakawa et al. in 2018.^44–46^ The Classical mevalonate pathway is generally utilized by mesophiles and eukaryotes and is the most well studied pathway.^47–49^ To our knowledge, only the Classical mevalonate pathway has been employed for IPP or DMAPP production via cell-free biocatalysis, with little-to-no optimization of the enzyme variants (Figure 1A).^29,34,50,51^ To evaluate whether enzymes sourced from thermophiles exhibit greater stability in cell-free reactions, resulting in enhanced reaction longevity and increased productivity, we identified a set of mevalonate pathway enzymes derived from organisms growing at temperatures exceeding 60 °C and compared them to the conventionally used mesophile-derived set of mevalonate pathway enzymes. Figure 1A depicts the newly selected thermophilic mevalonate pathway enzymes, which utilize the Archaea I pathway (Table S1). The thermophile-derived Archaea I set of enzymes was compared to the commonly used mesophile-derived Classical mevalonate pathway enzyme variants, originally reported by Korman et al.^29,51^ In summary, the thermophilic pathway exhibited a longer operating lifetime, produced higher product titers, and tolerated the presence of solvent-like molecules, each an indicator of a more robust and productive cell-free pathway.

**Figure 1:**
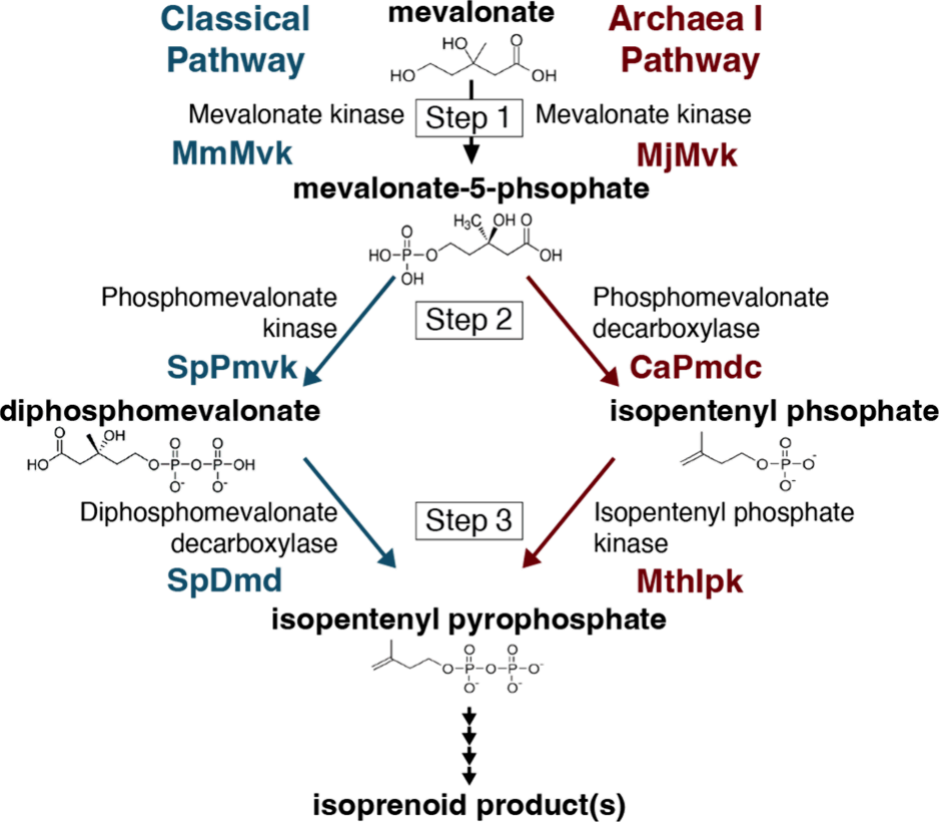
Comparison of Classical and Archaea I lower mevalonate pathways. The Classical pathway is shown in blue, first published by Korman et al., 2017.^29^ The Archaea I pathway is shown in red, depicting the newly selected thermophilic organism-derived enzymes (see Table S1 for enzyme details).

## Experimental (Materials and Methods)

### Expression and Purification of Enzymes

Genes encoding the enzyme of interest were cloned individually into a pET28 backbone, conferring Kanamycin resistance. See Tables S1–S2 for all genes cloned in this study. Plasmids were transformed into BL21­(DE3) *E. coli* (New England Biosciences) and plated at low density to grow individual colonies. A single colony was picked and seeded into a starter of Luria Broth (LB, FisherScientific), supplemented with 50 ug/mL Kanamycin (FisherScientific). The culture was scaled up with 1% volume of starter into 1 L of LB and then incubated at 37 °C and 225 rpm. Upon reaching an optical density at 600 nm (OD_600_) between 0.5 and 0.7, isopropyl β-D-1-thiogalactopyranoside (IPTG, Millipore-Sigma) was spiked into the culture to a final concentration of 1 mM to induce protein expression. The culture was returned to the incubator with the temperature lowered to 18 °C. After 20 h, the culture was centrifuged at 5000*g* for 10 min, and the supernatant was decanted; the pellet was frozen at −20 °C.

For enzyme purification, the cell pellet was suspended in HisA buffer (25 mM imidazole (Millipore-Sigma), 400 mM NaCl (FisherScientific), and 50 mM HEPES (Millipore-Sigma) at pH 7.5) and sonicated on ice. The sample was then centrifuged at 20,000*g* for 20 min, and supernatant was loaded onto an Akta Pure Chromatography (AKTA) system for His-trap purification with a 5 mL HisTrap FF (Ni-NTA resin, Cytiva) column. Elution was performed with HisB (300 mM imidazole, 150 mM NaCl, and 50 mM HEPES at pH 7.5). Eluted protein was directly loaded onto a Sephadex G-25 desalting column (Cytiva) to buffer exchange with GF buffer (50 mM HEPES at pH 7.5 and 100 mM NaCl). Eluted fractions were pooled, concentrated with Amicon 10KDa MWCO columns (Millipore-Sigma), and supplemented with glycerol (Millipore-Sigma) to a final concentration of 10%. Enzyme concentration was assayed by Bradford Plus Protein Assay Kit (Pierce), and then samples were aliquoted and flash frozen on liquid nitrogen before storage at −20 °C.

For visual inspection of enzymes, samples were mixed with 4× Bolt LDS sample buffer (ThermoScientific) and heated to 95 °C for 20 min before loading into Bis-tris 4%–10% WedgeWell precast gels (Invitrogen). Electrophoresis was performed in 3-(N-morpholino) propanesulfonic acid (MOPS, FisherScientific) buffer at 160 V for 40 min in the Invitrogen Mini Gel Tank. The gel was then stained using the Thermo Scientific Pierce Power Station, according to manufacturer’s instructions.

### Determination of Thermostability of Enzyme Variants

One hour heat treatment was conducted to probe enzyme thermostability. The enzyme sample was diluted to 1 mg/mL in GF buffer and then heat-treated for 1 h at various temperatures (4 to 70 °C) in a BioRad C1000 Touch Thermal Cycler. Enzyme thermostability was determined based on percent activity remaining comparing each heated sample to activity of the enzyme incubated at 4 °C. Activity was assayed using the “enzyme activity assays” method detailed below. Statistical analysis was conducted by *t* test comparing % activity remaining between the unheated, 4 °C sample, and the postheat sample for each temperature individually.

Additionally, the soluble protein remaining after heating was quantified. After heat-treatment, samples were centrifuged at 2,000*g* for 10 min to pellet insoluble enzyme. Remaining soluble enzyme was analyzed using the Bradford Assay. Qualitative verification was conducted by loading equal volumes from each sample on an SDS-Page gel to visualize the remaining soluble protein.

### Analysis of Mevalonate Generation

Similar to previous studies, mevalonolactone (Millipore-Sigma) was suspended in 0.8 mol equiv of NaOH and incubated at 30 °C overnight to allow for ring opening to mevalonate.^52^ After 24 h, more NaOH was added to bring the pH within the range of 7.5 and 8 before subsequent use. Data indicates approximately one-third of mevalonolactone converted to bioavailable mevalonate using this method (Figure S2, Table S3).

### Enzyme Activity Assays

All enzymes tested consume ATP, which permitted the universal use of a coupled assay with pyruvate kinase and lactate dehydrogenase (Pyk-Ldh mixture, Sigma-Aldrich) to translate ATP consumption to a decrease in NADH absorbance at 340 nm. Assay concentrations per 150 μL reaction included 0.5 mM ATP (Millipore-Sigma), 4 mM Phosphoenolpyruvate (Roche), 0.7 mM NADH (Fisher Scientific), 6 mM base-treated mevalonolactone (effectively 2 mM mevalonate), 0.75 μL of Pyk-Ldh, 80 mM HEPES pH 7.7, 15 mM MgCl_2_ (Millipore-Sigma), 5 mM KCl (Millipore-Sigma), and 0.5 mM MnCl (Millipore-Sigma). All assays were performed in triplicate (n = 3) using a SpectraMax190 plate reader. For the steps 2 and 3 reactions, previous steps were allowed sufficient time for completion; NADH was supplemented, and it was confirmed that no further reaction was ongoing before the target enzyme was added.

### Analysis of Limonene Synthesis

Using activity rates determined at 22 °C (Figure 3), enzyme loadings were calculated to match turnover rates for each step between the Classical and Archaea I pathways. Target mevalonate pathway enzymes were mixed with downstream limonene synthesis enzymes in buffer with 80 mM HEPES pH 7.7, 15 mM MgCl_2_, 5 mM KCl, and 0.5 mM MnCl. Table S2 details all enzyme and cofactor loadings. The 300 μL reactions were set up in quadruplicate (n = 4) in HPLC vials (Fisher Scientific) with screw tops (Fisher Scientific) to allow for daily sampling. The aqueous reaction was immediately overlaid with 500 μL of 2,2,4-trimethylpentane (TMP, Millipore-Sigma) spiked with β-pinene (1:500 dilution β-pinene:TMP, Millipore-Sigma) as an internal standard. Reactions were incubated at room temperature (22 °C) or in a hybridization oven (VWR) at 40 °C. Sampling was conducted by mixing 10 μL of the overlay with 290 μL of pure hexanes (Millipore-Sigma) and run on a GC (Agilent, 7890B) equipped with a J&W DB-5 ms column (30 m, 0.25 mm, 0.25 μm, 7-in. cage, Agilent) then quantified by MS (Agilent, 5977B). One μL of the sample was injected, then limonene and pinene were identified by retention time comparable to a standard curve. Helium was used as a carrier gas at a flow rate of 1 mL/min. The oven temperature started at 75 °C and was held for 5 min before ramping to 80 °C at a rate of 1 °C/min, then to 250 °C at a rate of 100 °C/min, and then held for 3 min to burn off any residual TMP. The retention window for β-pinene and limonene was 6.25 ± 0.45 and 8.4 ± 0.7 min, respectively (Figure S3 for representative trace of a standard of limonene (Thermo Scientific) and β-pinene).

## Results and Discussion

### Preliminary Selection of Optimal Thermophile-Derived Enzyme Variants

The mevalonate kinase from the thermophile *Methanocaldococcus jannaschii* (MjMvk) was selected as the optimal variant for the initial step of the thermophilic mevalonate pathway due to numerous reports in the literature and detailed *in vitro* characterization.^53–56^ Broader screening was conducted to identify optimal thermophilic biocatalysts, based on thermostability and activity at 22 °C, for steps 2 and 3 of the pathway (Figure 2). Phosphomevalonate kinase (SsPmvk) and diphosphomevalonate decarboxylase (SsDmd) from the archaeon *Saccharolobus solfataricus* (the only characterized thermophile that utilizes the Classical mevalonate pathway) were screened to determine thermostability and activity at 22 °C.^57,58^ For step 2, the SsPmvk surprisingly lacked stability at 50 °C, as a significant amount of enzyme precipitated during 1 h of heating (Figure 2A). This could be due to destabilizing buffer conditions. Thermal instability of an enzyme from a thermophilic organism has been previously documented, which can be due to exposure to foreign conditions such as buffer and pH, combined with thermal stress.^59^ Given that SsPmvk was essentially inactive at 22 °C (∼0.01 μmol ATP/min/mg), further thermostability characterization, such as activity postheating or SDS-page verification, was not conducted to fully confirm whether this variant was denaturing at 50 °C (Figure 2B). For step 3, the SsDmd was thermostable to 60 °C; however, its activity at 22 °C was negligible (<0.001 μmol ATP/min/mg, Figure 2C, D). Having tested the available thermophile-derived Classical pathway enzymes, we turned to the Archaea I mevalonate pathway enzymes to assemble a thermophile-derived lower mevalonate pathway.

**Figure 2:**
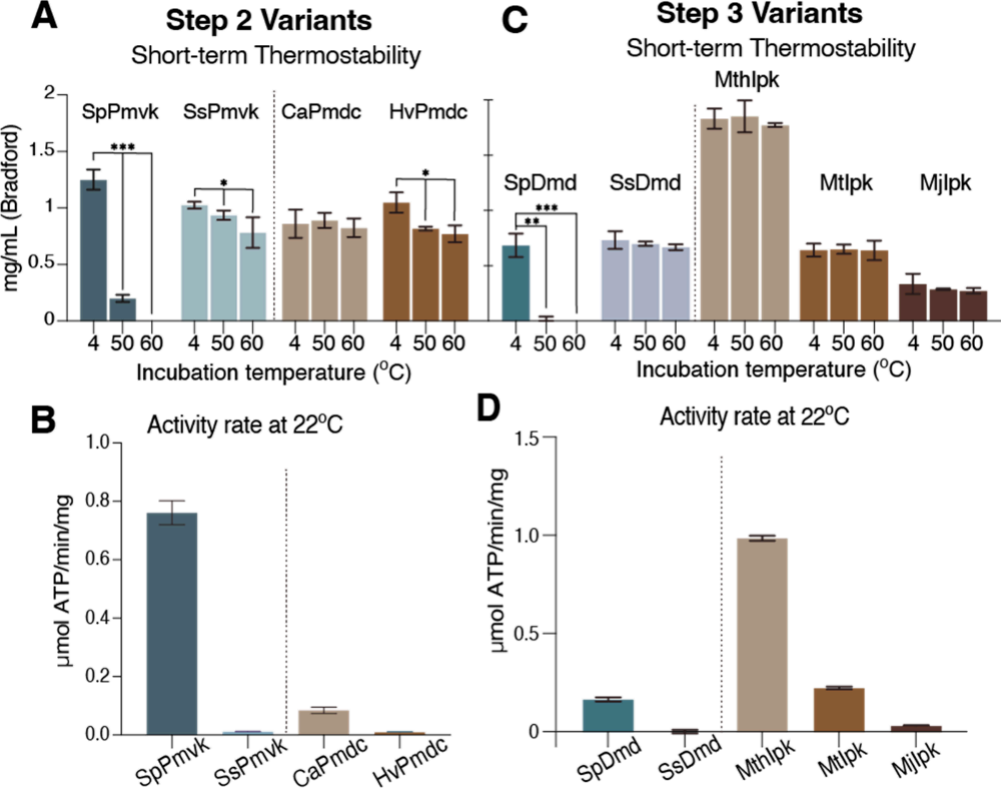
Broad variant screening was performed to find active and thermostable enzyme variants. A. Step 2 variants, Bradford assay of enzyme remaining soluble after 1 h heating. *t* tests were used to compare enzyme concentration between 4 °C and postheat samples individually. *p < 0.05, **p < 0.01, ***p < 0.001. B. Activity rate (μmol ATP/min/mg) of step 2 enzyme variants at 22 °C. C. Step 3 variants, Bradford assay of enzyme remaining soluble after 1 h of heating. *t* tests were used to compare enzyme concentration between 4 °C and postheat samples individually. *p < 0.05, **p < 0.01, ***p < 0.001. D. Activity rate of step 3 enzyme variants at 22 °C. (Dashed lines separate Classical and Archaea I pathway enzymes tested.)

For step 2, the previously characterized phosphomevalonate decarboxylase from *Haloferax Volcanii* (HvPmdc) was both unstable to 50 °C and nearly inactive at 22 °C (Figure 2A,B).^60^ The putative Pmdc from *Chloroflexus aurantiacus* (CaPmdc), however, was thermostable, and remained soluble (with no precipitation) at 60 °C. Additionally, CaPmdc was sufficiently active in decarboxylating phosphomevalonate at 22 °C (0.8 μmol ATP/min/mg, Figure 2B). For these reasons, CaPmdc was selected as the optimal variant for step 2 of the pathway. Lastly, isopentenyl phosphate kinases (Ipk) were screened for step 3 of the Archaea I pathway. Chen and Poulter previously compared *Methanothermobacter thermautotrophicus* (MthIpk) and *Thermoplasma acidophilum* (TaIpk). MthIpk was faster *k*_cat_ (27.5 s^–1^) than TaIpk (8 s^–1^) at 37 °C thus leading us to choose MthIpk for early screening.^61^ Another Ipk, from *Methanocaldococcus jannaschii* (MjIpk), was previously characterized.^62,63^ Additionally, we selected a new putative enzyme from Uniprot, *Methanosaeta thermophila* (MtIpk) due to a more modest growth temperature of the source organism, around 60 °C, as opposed to *M. jannachii* (85 °C) and *M. thermautotrophicus* (70 °C). We hypothesized that MtIpk would be faster at 22 °C due to lower evolved temperature optima as compared to enzymes from more hyperthermophilic organisms.^64^ Results of direct comparison between MjIpk, MthIpk, and MtIpk show all variants are thermostable to 60 °C (Figure 2C). However, MthIpk displayed superior activity at 22 °C (Figure 2D) compared to other Ipks tested. For this reason, MthIpk was selected as the final variant for the Archaea I pathway.

Having selected a new thermophile-derived pathway of enzymes, we systematically tested whether these enzymes would constitute a more reliable and productive pathway compared to the Classical pathway commonly employed in cell-free systems. The Classical pathway is composed of mesophile-derived enzymes including, mevalonate kinase from *Methanosarcina mazei* (MmMvk) and *Streptococcus pneumoniae* phosphomevalonate kinase (SpPmvk) and diphosphomevalonate decarboxylase (SpDmd), employed in two cell-free publications to date.^29,51^

### Comparison of Classical and Archaea I Pathway Enzyme Activity

One important factor for determining the efficiency of cell-free systems is the activity of the pathway of enzymes under operating conditions. Ambient tempearure (∼22 °C) is commonly used for cell-free reactions because cofactors remain stable for longer and to reduce the energy input required for heating.^65,15,66^

Initial activity rates at 22 °C were found to be faster for the Classical pathway enzymes as compared to Archaea I pathway enzymes when analyzing enzymes with similar reaction mechanisms (Figure 3). In comparing initial phosphorylation of mevalonate, MmMvk’s specific activity (2.04 ± 0.54 μmol ATP/min/mg) was found to be 3× that of MjMvk (0.59 ± 0.11 μmol ATP/min/mg). Interestingly, the order of reactions in the Classical and Archaea I pathways is reversed (Figure 1). In the Classical pathway, mevalonate-5-phosphate undergoes another phosphorylation, followed by a decarboxylation. The Archaea I pathway decarboxylates mevalonate-5-phosphate, then another phosphorylation occurs. When comparing enzyme rates for the second phosphorylation event, we found that SpPmvk (3.98 ± 0.13 μmol ATP/min/mg) was 1.3× faster than MthIpk (3.1 ± 0.12 μmol ATP/min/mg). Lastly, when comparing the decarboxylation reaction, the Classical pathway enzyme, SpDmd (0.33 ± 0.01 μmol ATP/min/mg) was 1.4× faster than the Archaea I pathway enzyme, CaPmdc (0.23 ± 0.01 μmol ATP/min/mg). The faster kinetics of the Classical, mesophilic-derived enzymes at 22 °C was an expected trend. Thermophile-derived enzymes have evolved to operate at much higher temperatures. For example, MjMvk and MthIpk experience temperature optima around 70 °C, close to the host organism’s growth optima.^53,61^ In cell-free systems, lower activity rates may be compensated for by loading higher amounts of enzyme. While employing more biocatalysts increases process costs, this can be compensated by screening for well-expressing enzymes or identifying enzymes with superior operating lifetime.

**Figure 3:**
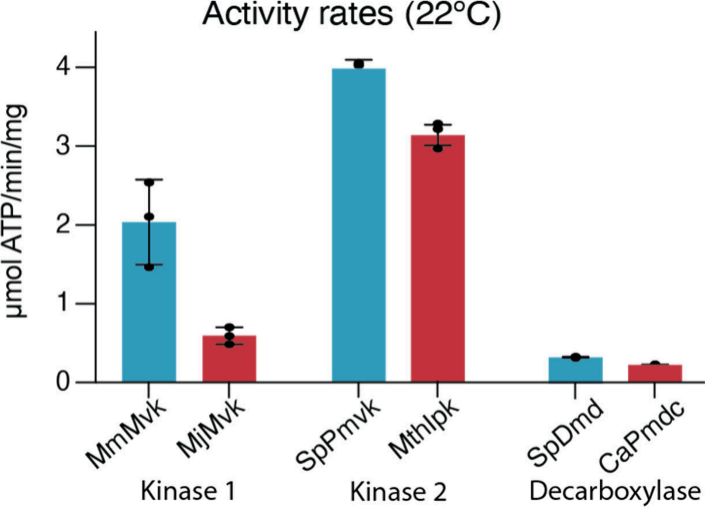
Enzyme activity rates (μmol ATP/min/mg enzyme) assayed at 22 °C. Enzyme rates between similar chemical reaction mechanisms are plotted side-by-side.

### Thermostability and Activity Retention

To characterize the thermostability of the enzyme variants, specific activity at 22 °C was measured after incubation at various temperatures for 1 h (Figure 4A–C). Enzyme loss due to precipitation was determined by Bradford assay and SDS-Page gels to inform whether activity loss was due to unfolding and precipitation (Figure S4, Figure S5). MmMvk was unexpectedly thermostable, with no significant activity loss detected at 60 °C, despite originating from a mesophilic archaeon (Figure 4A).^67^ However, both the Bradford assay and SDS-Page gels indicated depletion of soluble protein starting at 60 °C, suggesting that this temperature was close to the thermostability limit of the protein. Phylogenetic assessment of MmMvk conducted by Primak et al., revealed a close common ancestor between MmMvk and a Mvk from *Pyrococcus Abyssi*, which grows at 96 °C. Our results suggest some stabilizing characteristics may be shared in MmMvk, making it thermostable to 50–60 °C.^52,68^ SpPmvk had an unexpected increase in activity after incubation at 30 and 40 °C; however, here there was no change in soluble protein assayed by Bradford, and the SDS-Page gel indicated loss of soluble protein after heating to 40 °C; this phenomenon was not further interrogated (Figure S4B, Figure S5B). Upon incubation at 50 °C, activity and soluble enzyme concentration dropped significantly, suggesting that the short-term thermostability limit lies between 40 and 50 °C (Figure 4B, Figure S4B). Of the Classical pathway enzymes, SpDmd was particularly unstable, with significant activity loss and soluble enzyme depletion found after incubation at 30 °C (Figure 4C, Figure S4C, Figure S5C). All of the Archaea I pathway enzymes were thermostable, with no significant activity loss, and minimal precipitation detected, to at least 60 °C (Figure 4A–C, Figure S4, Figure S5). Taking into account these thermostability results and the activity rates at 22 °C, there is a clear trade-off between activity and stability of the enzymes.^69,70^ Specifically, the Classical pathway enzymes are more active at lower temperatures, correlating to the propensity for unfolding and aggregation at elevated temperatures. The converse was observed for the Archaea I pathway, composed of thermostable enzymes; while activity was reduced at 22 °C, greater stability is observed at increased temperatures.

**Figure 4:**
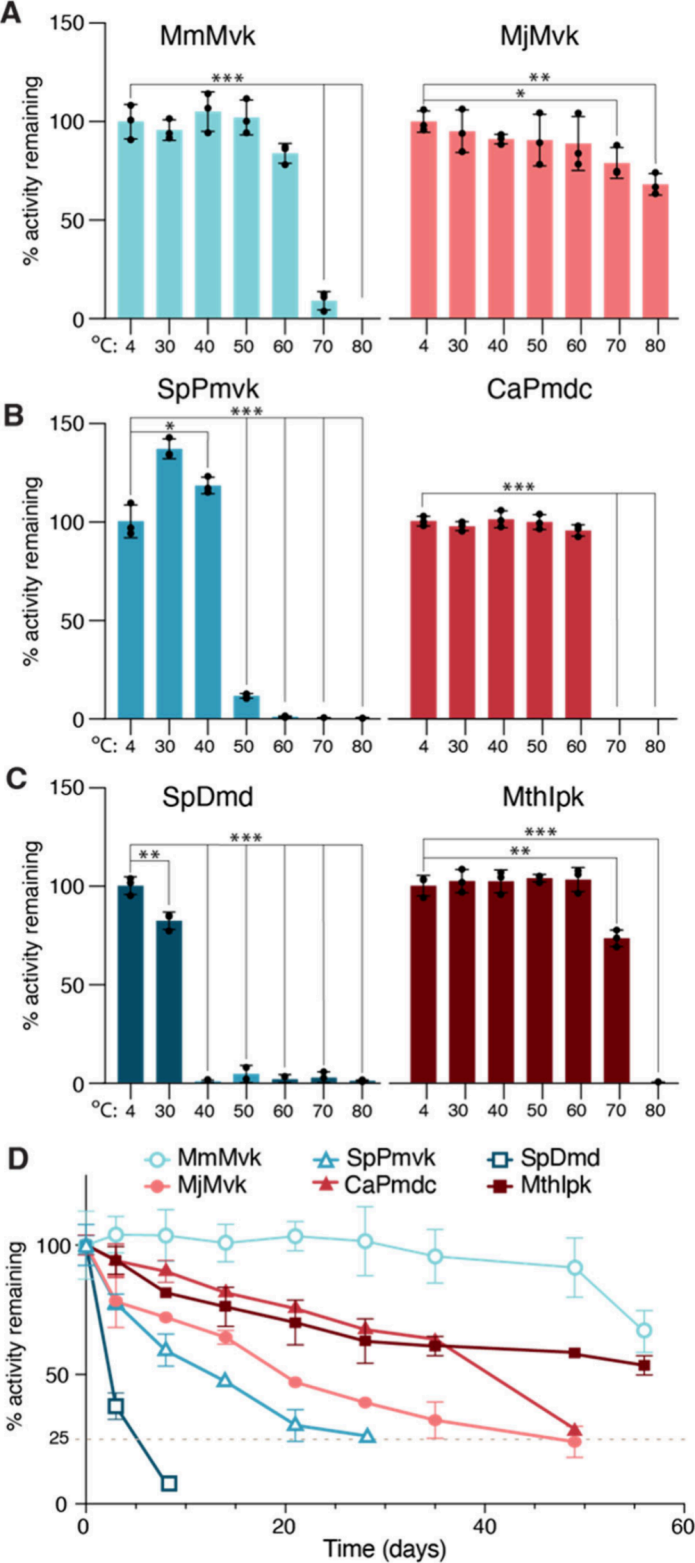
Probing stability of enzyme variants. A–C. Quantification of enzyme activity after 1 h of incubation at various temperatures. Values reported are % activity of enzyme postheat compared to 4 °C incubated samples. *t* tests compared between 4 °C incubated and each higher temperature as individual comparisons, *p < 0.05, **p < 0.01, ***p < 0.001. D. Long-term activity retention of enzymes at 22 °C, reported as % activity remaining relative to initial time point. Samples were incubated over 2 months with activity assayed at various time points.

We hypothesized that thermostability would correlate with a long-term operating lifetime at a modest temperature. To assess operating lifetime of the enzymes, each enzyme was individually incubated at 22 °C, and its activity retention was measured over time (Figure 4D). The thermostable enzymes generally exhibited longer activity retention. Over the two-month testing period, MthIpk retained >50% activity. CaPmdc and MjMvk were less stable, losing approximately 75% activity after 49 days. In contrast, the most unstable enzyme in the Classical pathway, SpDmd, lost the same amount of activity (75%) within only 8 days. SpPmvk lost 75% activity within 28 days. Consistent with its high thermal stability, the Classical pathway enzyme, MmMvk, retained >65% activity over the entire testing period. Notably the MmMvk retained activity for longer than its thermophile-derived counterpart, MjMvk. In the future, directly comparing MmMvk vs MjMvk in isoprenoid production reactions would reveal which enzyme is more productive under reaction conditions. A cell-free pathway is limited by the most unstable enzyme, and flux through the pathway will halt if one enzyme loses activity. Based on the enzyme activity retention results, the thermostable Archaea I pathway demonstrated a 6-fold improvement in operating lifetime of the ensemble pathway. To visualize the relation between thermostability and activity retention,% activity retained for each variant at 14 days was plotted against the long-term activity retention at 22 °C. The data indicate a positive correlation with an R-squared of 0.88 between thermostability and long-term activity retention (Figure S6). Having confirmed the thermal and temporal stability of the thermophile-derived enzymes, we next evaluated whether these enzymes would constitute a more productive pathway for the synthesis of limonene, a model monoterpene product.

### Comparison of Limonene Production by the Classical and Archaea I Pathways

To determine whether the use of thermophilic enzymes in the lower mevalonate pathway enhances productivity in cell-free reactions, limonene production was compared between the previously employed mesophile-derived Classical pathway enzymes and the newly assembled thermophile-derived Archaea I mevalonate pathway (Figure 5). Reactions were prepared such that turnover at each step between the Classical and Archaea I pathway enzymes matched based on initial activity rates determined for each enzyme at 22 °C. Enzyme concentration in the reaction was tuned to match a turnover of 0.31 μmol/min to ensure that the downstream limonene production enzymes (isopentenyl-d-isomerase (Idi), geranyl diphosphate synthase (GppS^71^), limonene synthase (LimS)) would not be rate limiting in reaction. An excess of Idi, Gpps, and LimS enzymes were added, ensuring turnover from these enzymes was >30 x faster than the mevalonate pathways (Table S2). The reaction converting mevalonate into limonene is highly ATP dependent (6 ATP molecules consumed per 1 limonene molecule produced). ATP recycling was maintained with creatine kinase and creatine-phosphate as a sacrificial substrate, again in excess. The limonene production reactions were intentionally loaded with low mevalonate pathway enzyme concentrations to minimize substrate or product inhibition and probe long-term reaction lifetime, rather than maximizing limonene production titers.

**Figure 5:**
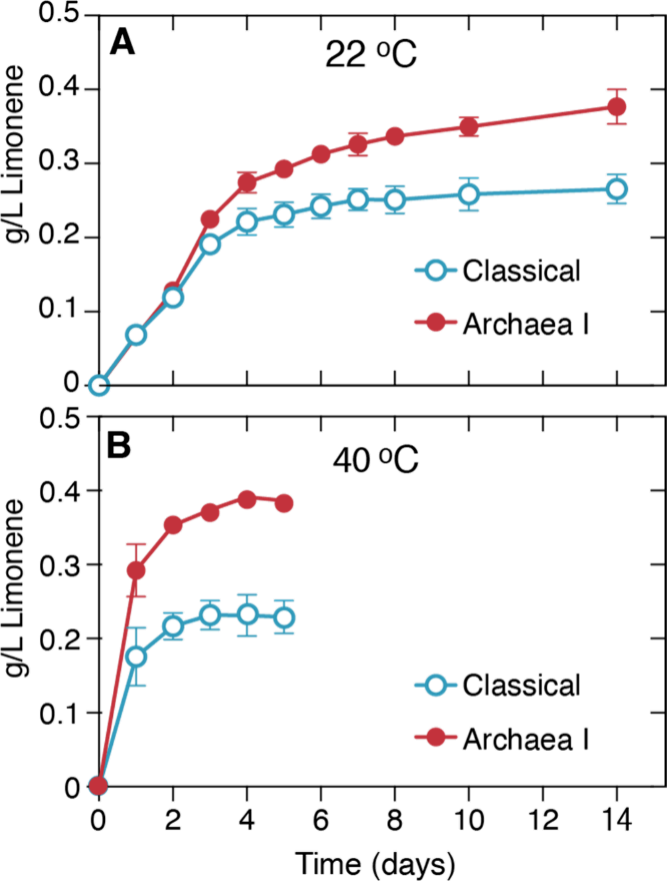
Limonene production by Classical or Archaea I mevalonate pathways. A. Limonene production at 22 °C, assayed over 14 days. B. Limonene production at 40 °C, sampling ceased when no further limonene was produced. Table S2 details the enzyme and substrate concentrations in these reactions.

At 22 °C, initial limonene production rates are equal for the first two days between the Classical and Archaea I pathways (Figure 5A). This suggests that the enzymes are behaving as predicted by the initial rate experiments. However, by day 3, the Archaea I pathway begins to outperform the Classical pathway (Figure 5A). By about day 6, the Classical pathway has ceased limonene production, whereas the Archaea I limonene titers continue to increase until day 14. These results indicate that the Archaea I pathway is productive for longer and able to achieve higher product titers. Of note, the limonene production rates did not reach the full potential expected based on kinetics for individual-enzyme assays. This could be due to substrate or product inhibition, for example MjMvk and SpPmvk have been shown to be inhibited by geranyl diphosphate or diphosphomevalonate and phosphomevalonate, respectively.^53,72^ Inhibitory molecules have not been characterized for the rest of the pathway enzymes, with the exception of MmMvk, which was found to be insensitive to substrate or product inhibition.^52^ However, it is unlikely that substrate or product inhibition hampers productivity until one or more of the pathway enzymes starts to degrade, at which point intermediates of the pathway start to accumulate. The production rates between the pathways at 22 °C were equal for the first 2 days, suggesting well-matched flux through the pathways without inhibitory effects impacting enzyme kinetics. Other potential inhibitory molecules, creatine and creatine phosphate, have not been characterized for their impact on the enzyme kinetics of these pathways and may contribute to reduced overall productivity. Another limiting factor may be the presence of the organic solvent overlay used to extract limonene continuously throughout production. It is known that enzymes tend to denature at interfaces, which may cause faster inactivation than demonstrated by our activity retention assays.^73–75^ It is likely that experimental iteration could further increase productivity of the system through tuning ATP recycling, altering product extraction protocols, and adjusting enzyme loading ratios to increase pathway productivity.

Running cell-free reactions at elevated temperature has the advantage of accelerating enzyme kinetics, however with the trade-off of faster enzyme denaturation or cofactor degradation. Limonene production at 40 °C was tested using identical reaction mixtures to those described above (Figure 5B). These reactions showed a remarkable rate increase. At 2 days, both mevalonate pathway reactions achieved limonene titers compared to 4–5 days at 22 °C. The Archaea I pathway began to outperform the Classical enzymes within the first 24 h and, by day 4, achieved limonene titers of 1.7-fold higher than those of the Classical pathway. Together, these results indicate that thermostable enzymes have two main benefits: they can withstand longer production reactions to achieve higher titers and tolerate elevated temperatures to enable faster reaction kinetics. The enzymes required for limonene production (Idi, Gpps, LimS, and Creatine kinase) were not thoroughly characterized for thermostability, which may be the cause for limited reaction lifetime at this elevated temperature.

### Analysis of Biocatalyst Production Burden

Although the Archaea I pathway generated higher limonene titers overall, a higher overall concentration of enzyme was required to match turnover rates with the Classical pathway enzymes. The Archaea I pathway required nearly two times more enzyme to match the rates of the Classical pathway (1.96 mg L^–1^ Archaea I pathway vs 1.15 mg L^–1^ for Classical, Figure S7A). This presents an increased burden associated with enzyme production, a major consideration of cell-free systems.^76,77^ To analyze the enzyme production burden between these pathways, we compared expression enzyme levels under a fixed set of conditions. Qualitatively, the Archaea I pathway enzymes were more enriched in *E. coli* lysates than the Classical pathway enzymes as visualized by SDS-page (Figure S7B). This favorable expression profile of the Archaea I pathway resulted in half of the amount of *E. coli* expression volume needed to produce enzymes for the Archaea I pathway compared to the Classical pathway (Figure S7C). These results underscore the importance of considering multiple factors in enzyme selection for cell free systems; slower reaction rates can be compensated for by increased enzyme production, which can be mitigated by sourcing well-expressing enzymes or optimizing enzyme production protocols. Future work in optimizing expression parameters, such as expression temperature and IPTG concentration, may result in even higher enzyme titers to further reduce biocatalyst production burden. Altogether, in limonene production reactions the thermophile-derived pathway both required less enzyme expression culture, and produced more product, demonstrating a marked improvement in lower mevalonate pathway efficiency.

### Effect of Solvent on Archaea I and Classical Pathways

There is considerable interest in employing cell-free systems for conversion of substrates other than glucose (e.g., ethanol) or formation of products (e.g., isoprenol) with enzyme-denaturing properties.^56,78^ For example, Liu et al. described a cell-free system linking modules producing Acetyl-CoA from ethanol, as well as conversion of mevalonate to isoprenol in a single pot.^56^ This poses a problem for cell-free systems since the presence of solvents promotes enzyme denaturation, ultimately reducing pathway flux.^79^ Several enzyme engineering campaigns have found a trend between increased thermostability and higher solvent tolerance.^78,80–82^ We hypothesized this correlation between thermostability and solvent-tolerance would apply to the thermophile-derived enzymes of the lower mevalonate pathway.

Activity retention of the ensemble Classical and Archaea I mevalonate pathways was compared in the presence of varying concentrations of ethanol or isoprenol, which serve as representative solvent-like substrates or products (Figure 6). For both ethanol and isoprenol, the Archaea I pathway retained a higher activity than the Classical pathway. This was apparent within the first 10 min, in which the Classical enzymes had lower activity rates upon exposure to solvent. Remarkably, the Archaea I pathway retained the same activity between the 0% and 25% (v/v) ethanol condition throughout the experiment (Figure 6A). In contrast, the Classical pathway lost 50% of its activity immediately upon exposure to 25% ethanol. Isoprenol was found to be more denaturing than ethanol, therefore lower concentrations were tested (Figure 6B). The Archaea I pathway tolerated all isoprenol concentrations tested for 24 h, after which activity rapidly decreased to less than 50% by day 3. In contrast, the Classical pathway steadily lost activity for both isoprenol concentrations, losing 75% or more activity within the initial 24 h. These trends in solvent stability indicated that the Archaea I, thermophile-derived pathway, is more tolerant to the presence of organic solvents, expanding potential applications of this pathway to new substrates or target products.

**Figure 6:**
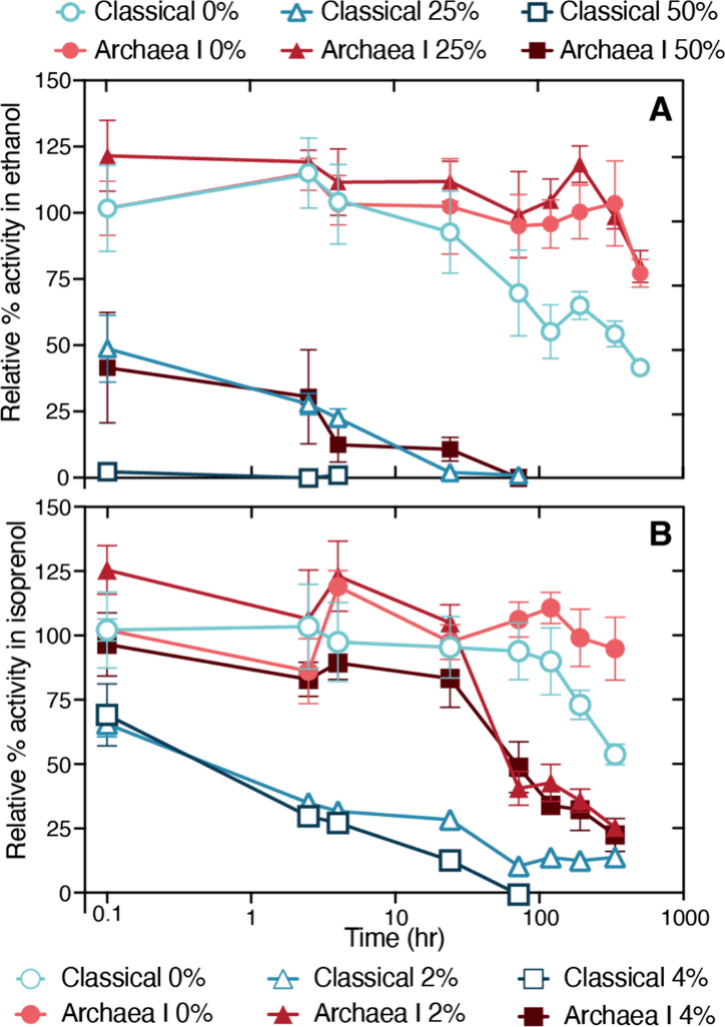
Solvent tolerance of the Classical and Archaea I pathways. A. Mixtures of pathways were suspended in various concentrations of ethanol, 0–50% (v/v), and assayed for % activity retained relative to 0% ethanol at the initial time point for the ensemble activity of the full pathway (steps 1–3). Activity was measured by an ATP-consumption assay of the ensemble pathway. B. Like A, enzymes were exposed to 0–4% isoprenol.

## Conclusions

Improving the stability of enzymes in cell-free reactions can help maximize the benefits of cell-free biocatalysis, thereby facilitating environmentally friendly chemical production.^83^ This work demonstrates an improvement in the stability of the lower mevalonate pathway by utilizing enzymes sourced from thermophilic organisms to enable more productive biocatalysis. Careful selection of thermophilic enzymes resulted in a 1.7× higher limonene production as compared to previously employed mesophilic enzymes. Here, limonene was used as an example target product; however, the principles for making cell-free reactions more robust, described here, have much broader implications. For example, IPP produced from this mevalonate module can be upgraded to a wide range of high-value isoprenoid products by swapping out the downstream enzymes specific to the product of interest (Figure S1).^18^ Altogether, this work addresses green chemistry principles of waste prevention (no byproduct formation, higher titer enzyme production), minimizing toxic materials (toxic solvents are not necessary to the process), and enhancing energy efficiency (no high temperatures required).^84^ This more efficient mevalonate pathway can sustainably produce higher titers of isoprenoids, requires lower energy input, and does not depend on heavy-metal catalysts, in contrast to traditional chemical catalysis.^10,12,13^ Additionally, with improved extraction methodologies or bioreactor design, the use of toxic solvents can be avoided. Lastly, sustainable on-demand isoprenoid synthesis bypasses challenges associated with current production pipelines, such as uncertainty in biomass availability and energy-intensive extraction methods.^8,9^

Beyond improving a particular cell-free pathway module, we believe that the strategies illustrated here can serve as a case study in the careful selection of natural variants when building cell-free pathways. The results of this study support previously hypothesized correlations that thermostability extends beyond enzyme tolerance to thermal stress, as demonstrated by its correlation to both long-term activity retention and solvent stability. To conclude, this work demonstrates that sourcing biocatalysts from thermophiles is advantageous for identifying optimal biocatalysts capable of withstanding the unique challenges encountered in cell-free systems.

## Supplementary Material



## Notes

Table S1: Enzyme details for variants in this study; Table S2: Mevalonate pathway and downstream enzyme loadings utilized for limonene experiments; Table S3: Determination of mevalonolactone conversion to mevalonate by base-treatment; Figure S1: Schematic of downstream pathways and chemicals built from isopentenyl diphosphate building blocks; Figure S2: Determination of treated-mevalonolactone conversion to mevalonate; Figure S3: Example GC/MS spectra from limonene synthesis experiments; Figure S4: Bradford assay quantification of protein remaining in solution after heating at various temperatures for 1 h; Figure S5: SDS-Page gels of enzyme remaining in solution postheat treatment; Figure S6: Quantifying the correlation between thermostability and% activity retention at 22 °C; Figure S7: Quantification of expression culture needed to produce enzyme for 1 L of cell-free reaction (PDF)
