# Supplementary material for: Thermostable Enzyme Variants in the Lower Mevalonate Pathway Improve Isoprenoid Production by Cell-Free Biocatalysis
Source: ACS Sustain Chem Eng. 2025 Aug 6;13(32):12971–80. doi: 10.1021/acssuschemeng.5c03763 (PMC12365920; doi:10.1021/acssuschemeng.5c03763)
Supplement: Supplementary file 1 [file sc5c03763_si_001.pdf]

SUPPORTING INFORMATION

Thermostable enzyme variants in the Lower Mevalonate Pathway improve isoprenoid production by cell-free catalysis

Sylvia A. Sarnik[1,2], Mia R. Martinsen[2,3], Tyler P. Korman[4], Daniel K. Schwartz[1], Joel L. Kaar[1], Yannick J. Bomble[2*]

[1]Department of Chemical and Biological Engineering, University of Colorado, Boulder, CO 80309, USA

[2]Biosciences Center, National Renewable Energy Laboratory, Golden, CO 80401, USA

[3]Department of Chemical Engineering, Colorado School of Mines, Golden, CO 80401, USA

[4]Exozymes, Inc., Monrovia, CA 91016, USA

**Corresponding Author:*
Yannick Bomble
National Renewable Energy Laboratory
Biosciences Center
Tel: 303-384-7729
Email: yannick.bomble@nrel.gov

Number of pages: 10
Number of tables: 3
Number of figures: 7

Table of contents

- Table S1- page 2

- Table S2- page 3
- Table S3- page 4
- Figure S1- page 5
- Figure S2- page 5
- Figure S3- page 6
- Figure S4- page 7
- Figure S5- page 8
- Figure S6- page 9
- Figure S7- page 10

Table S1: Enzyme details for variants in this study. **A.** Classical pathway enzymes employed by Korman et al. **B**. Newly selected alternative pathway enzymes. **C**. Other screened enzymes for steps 2 and 3 of the mevalonate pathway.

	**Enzyme**	**Source Organism**	**Catalytic activity**	**Uniprot ID/ Accession #**
**A. Selected Classical mevalonate pathway enzymes**				
Step 1	MmMvk	*Methanosarcina mazei*	Mevalonate kinase	Q8PW39
Step 2	SpPmvk	*Streptococcus pneumoniae*	Phosphomevalonate Kinase	Q8DR49
Step 3	SpDmd	*Streptococcus pneumoniae*	Diphosphomevalonate Decarboxylase	Q8DR50
**B. Selected Alternative mevalonate pathway enzymes**				
Step 1	MjMvk	*Methanocaldococcus jannaschii*	Mevalonate kinase	Q58487
Step 2	CaPmdc	*Chloroflexus aurantiacus*	Phosphomevalonate Decarboxylase	A9WEU8
Step 3	MthIpk	*Methanothermobacter thermautotrophicus*	Isopentenyl Phosphate Kinase	O26153
**C. Other screened enzyme variants**				
Step 2	SsPmvk	*Saccharolobus solfataricus*	Phosphomevalonate Kinase	WP_009992639.1
Step 2	HvPmdc	*Haloferax volcanii*	Phosphomevalonate Decarboxylase	D4GXZ3
Step 3	SsDmd	*Saccharolobus solfataricus*	Diphosphomevalonate Decarboxylase	Q97UL5
Step 3	MthIpk	*Methanothermobacter thermautotrophicus*	Isopentenyl Phosphate Kinase	O26153
Step 3	MtIpk	*Methanosaeta thermophila*	Isopentenyl Phosphate Kinase	A0B6E2

Table S2: Mevalonate pathway and downstream enzyme loadings utilized for limonene experiments, conducted at 300µL scale in quadruplicate vials. Creatine-kinase and creatine-phosphate were used to conduct ATP recycling. U/mg for downstream enzymes (Idi, Gpps, LimS) were identified by Korman et al. 2017.

**Target pathway enzymes**	**U/mg (µmol/min/mg)**	**mg/L loading**	**rate (µM/min)**
MmMvk	2.04	0.15	0.31
SpPmvk	3.98	0.077	0.31
SpDmd	0.33	0.92	0.31
MjMvk	0.59	0.52	0.31
CaPmdc	0.23	1.35	0.31
MthIpk	3.13	0.097	0.31

**Other Enzymes**	**U/mg (µmol/min/mg**	**mg/L loading**	**turnover rate (µM/min)**
E. coli Isopentenyl-D-isomerase (thermostable variant) (Idi)	4.3	100	430
Geobacillus stearothermophilus Geranyl diphosphate synthase (GppS) (FPPS S82F variant) [1]	7.3	100	730
Mentha spicata Limonene synthase (LimS) (thermostable variant)	0.01	1200	12
Rabbit Muscle Creatine Kinase	350	180	63000

**Cofactors**	**mM**
Mevalonolactone (base pre-treated)	125
Adenosine Triphosphate	2
Creatine-phosphate	125

[1] Ohnuma S, Nakazawa T, Hemmi H, et al. Conversion from farnesyl diphosphate synthase to geranylgeranyl diphosphate synthase by random chemical mutagenesis. *J Biol Chem*. 1996;271(17):10087-10095. doi:10.1074/jbc.271.17.10087

Table S3: Determining mevalonolactone conversion to mevalonate by base-treatment. Various amounts of mevalonolactone pretreated with base (see methods, column 1) were added at to a mevalonate kinase reaction to monitor phosphorylation of mevalonate through a linked assay looking at ATP-consumption with pyruvate kinase-lactate dehydrogenase. The reaction was allowed to come to completion while monitoring absorbance at 340nm (A340, **Figure S2**). Amount of NADH consumed (column 2) was calculated as the difference in A340 signal between 0mM mevalolactone input and the final A340 signal for each input mevalonolactone concentration. This was used to calculate what % of mevalonolactone becomes the bioavailable mevalonate form which mevalonate kinase can phosphorylate (column 3). These values consistently show 30-33% conversion from the lactone to the mevalonic acid from.

**nmol mevalolactone input**	**nmol NADH consumed**	**% of mevalonolactone utilized**
0	0	0
0.06	0.0174	29
0.0975	0.033	33.8
0.15	0.0474	31.6
0.225	0.0747	33.2

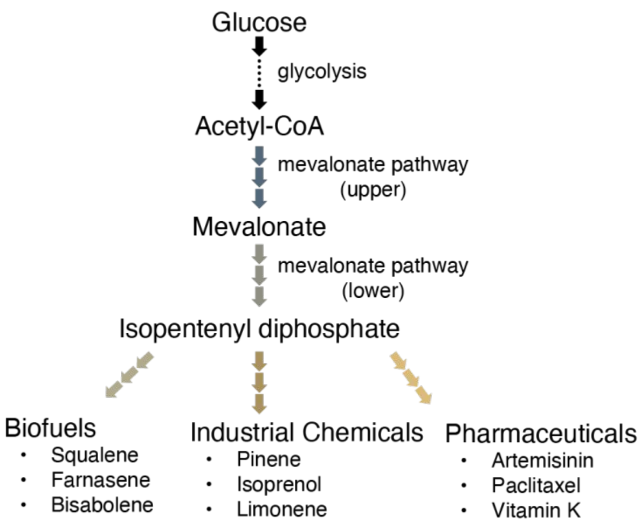

Figure S1: Schematic of downstream pathways and chemicals built from isopentenyl diphosphate (IPP) building blocks.

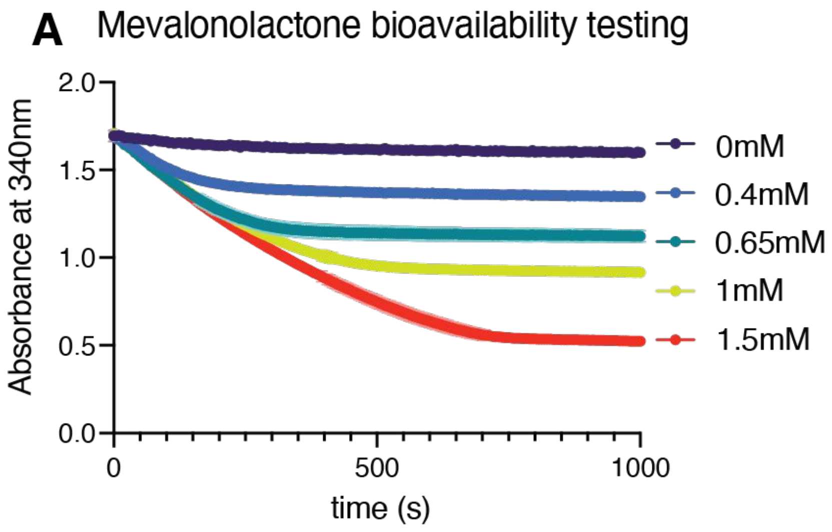

Figure S2: Determining treated-mevalonolactone conversion to mevalonate. Mevalonolactone pretreated with base (see methods) was added at various concentrations to a mevalonate kinase reaction and allowed to come to completion while monitoring absorbance at 340nm (A340). Calculations are found in **Table S3**.

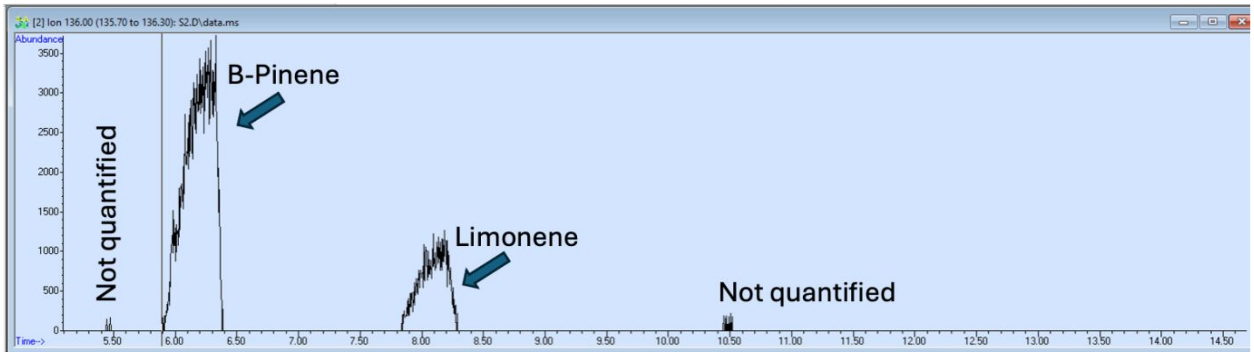

Figure S3: Example GC/MS spectra from limonene synthesis experiments. A standard sample of Limonene at 0.5g/L and β-Pinene spiked in at same levels of experimental samples. This trace was processed by extracting ion chromatograms for 132 m/z +/- 0.3, the maximum expected size for both molecules. Samples were quantified based on these peaks as well.

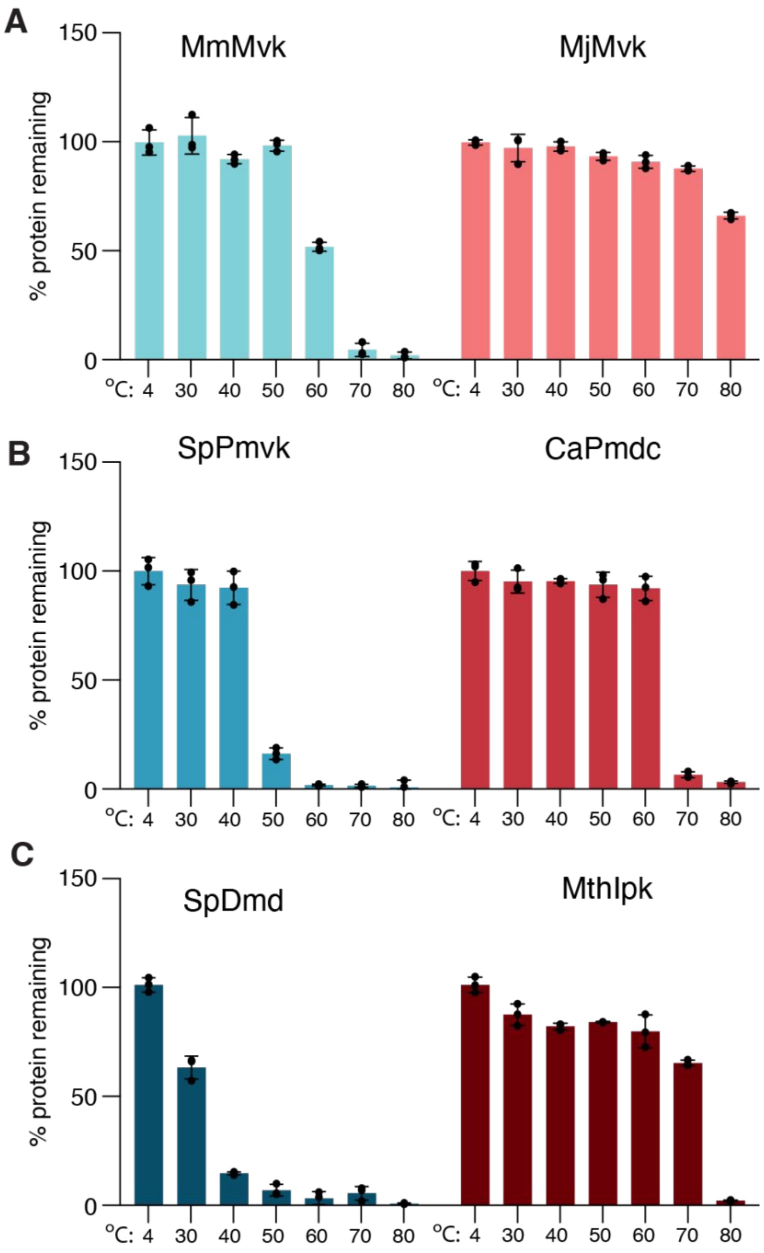

Figure S4: Bradford assay quantification of protein remaining in solution after heating at various temperatures for 1 hour. **A**. Step 1, mevalonate kinase comparison, MmMvk (left) and MjMvk (right). **B**. Step 2, comparison of phosphomevalonate kinase SpPmvk (left) vs. phosphomevalonate decarboxylase CaPmdc (right). **C**. Step 3, comparison of diphosphomevalonate decarboxylase SpDmd (left) vs. isopentenyl phosphate kinase MthIpk (right).

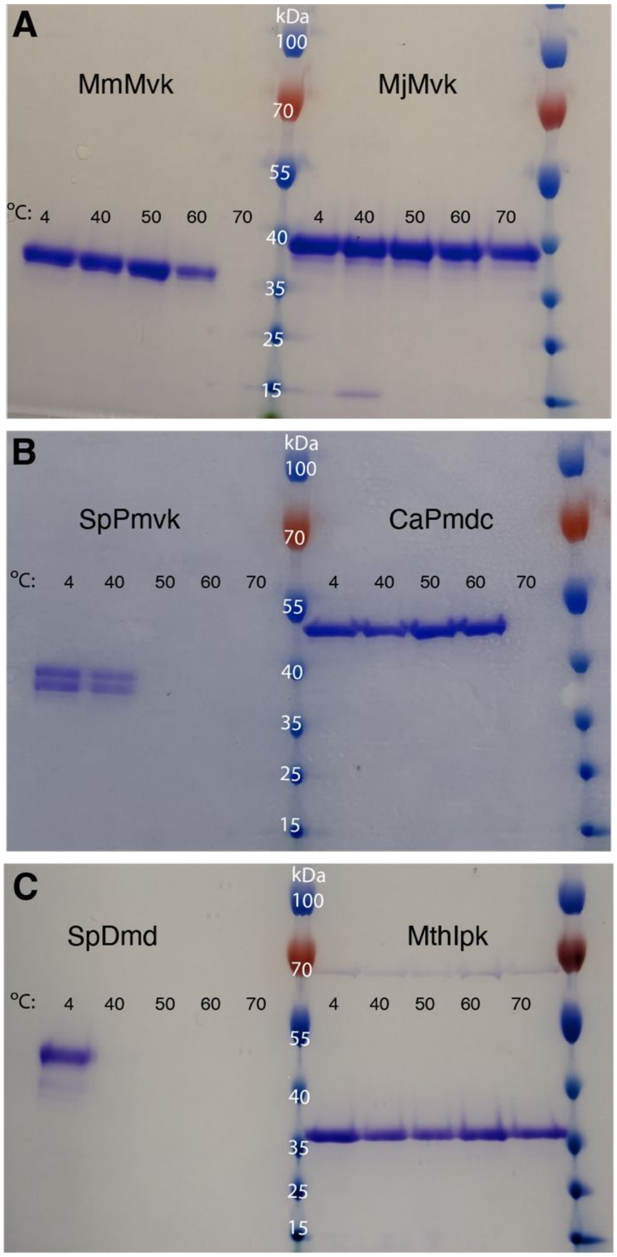

Figure S5: SDS-Page gels of enzyme remaining in solution post-heat treatment. Equal volume of soluble protein remaining post-heat was loaded on the SDS-Page gel. Disappearance of a protein band indicates instability and precipitation of that enzyme at the indicated temperature. **A**. Step 1, Mevalonate kinase comparison, MmMvk (left) and MjMvk (right). **B**. Step 2, comparison of phosphomevalonate kinase SpPmvk (left) vs. phosphomevalonate decarboxylase CaPmdc (right). **C**. Step 3, comparison of diphosphomevalonate decarboxylase SpDmd (left) vs. isopentenyl phosphate kinase MthIpk (right).

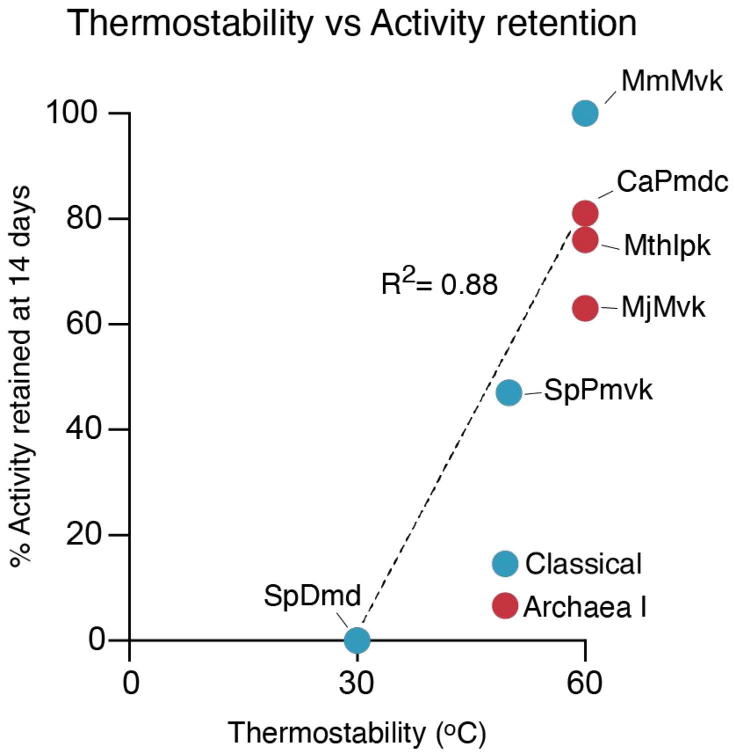

Figure S6: Quantifying the correlation between thermostability and % activity retention at 22°C. Thermostability was determined as maximal temperature at which no significant activity loss was found, plotted against % activity retained at 14 days.

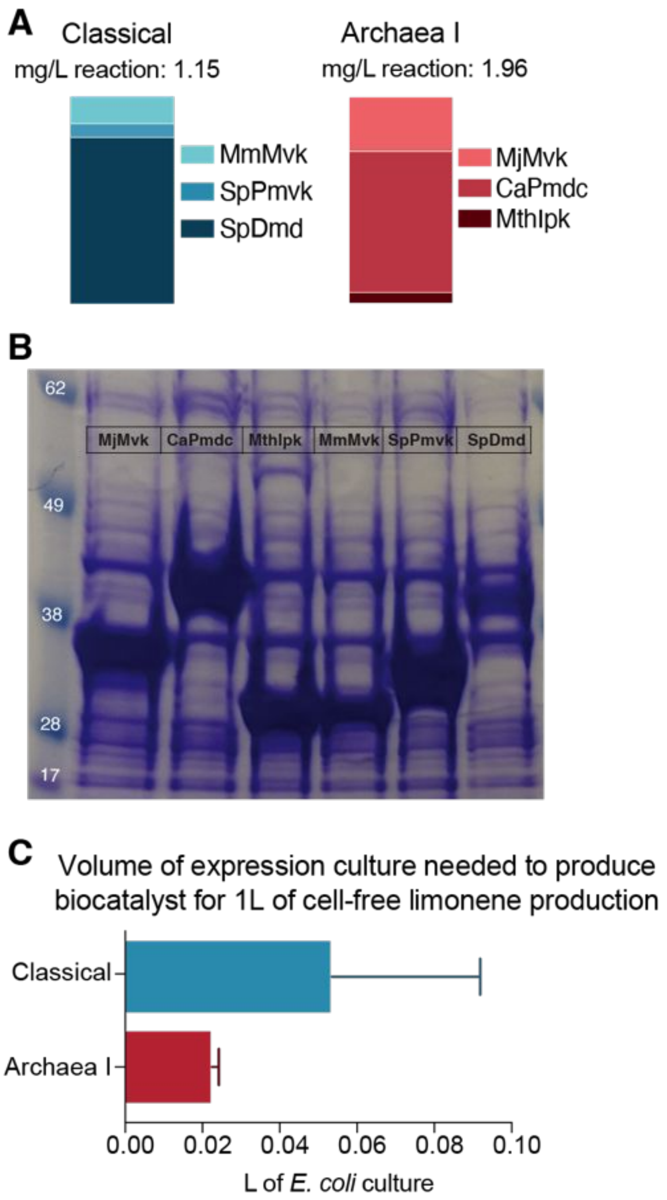

Figure S7: Quantification of expression culture needed to produce enzyme for 1 L of cell-free reaction. **A.** Quantification of total amount of enzyme added to limonene production experiments (See **Table S2** for exact loadings of each enzyme). **B.** Equal volumes of lysate of *E. coli* expression cell lines were loaded in the SDS-PAGE. Left three lanes show the Archaea I pathway lysates, strongly enriched for the overexpressed target protein. Right three lanes show the classical pathway enzymes, with steps 1 and 3 (MmMvk, lane 5 and SpDmd, lane 7) being the least overexpressed enzymes (qualitatively). C**.** Extrapolation determining the liters of *E. coli* expression culture needed to produce biocatalyst for 1 L of a cell-free limonene production reaction. Calculations were based on enzyme loading in limonene production reactions (A), and enzyme yields post his-tag purification from 1 L *E. coli* flask expressions (data not shown).
